# Negative Emotion Regulation Self‐Efficacy Moderates the Association Between PTSD Symptom Severity and Suicidal Thoughts and Behaviors in a Trauma‐Exposed Community Sample of Adults

**DOI:** 10.1002/jclp.70046

**Published:** 2025-09-29

**Authors:** Kayla E. Hall, Matthew T. Tull, Kim L. Gratz

**Affiliations:** ^1^ Department of Psychology University of Toledo Toledo Ohio USA; ^2^ Department of Psychological & Brain Sciences Texas A&M University College Station Texas USA; ^3^ Lyra Health Burlingame California USA

**Keywords:** emotion regulation, PTSD, trauma, self‐efficacy, suicide, suicide risk

## Abstract

Individuals who experience symptoms of post‐traumatic stress disorder (PTSD) are more likely to experience suicidal thoughts and behaviors (STB). However, additional research is needed to clarify for whom the relationship between PTSD symptoms and STB is the strongest. Considering that both PTSD and STB are characterized by difficulties with emotion regulation, one construct worth exploring in this regard is emotion regulation self‐efficacy (ERSE), or one's beliefs in their ability to regulate negative emotions. Thus, the current study sought to explore the role of ERSE in the association between PTSD symptoms and STB. Using Amazon's Mechanical Turk, participants (*N* = 227; *M*_age_ = 39.91, 65.2% assigned female sex at birth) completed self‐report measures of PTSD symptoms, ERSE, and STB. Controlling for depression, results revealed a significant interaction between PTSD and ERSE in relation to STB. Simple slopes analyses revealed a significant positive association between PTSD symptom severity and STB among individuals with low or moderate, but not high, self‐efficacy for managing negative emotions. Results highlight the relevance of ERSE in understanding STB among individuals experiencing PTSD symptoms.

Post‐traumatic stress disorder (PTSD) is a trauma‐ and stressor‐related disorder associated with reexperiencing symptoms, negative alterations in cognition and mood, avoidance behavior, and hyperarousal symptoms that persist after exposure to a traumatic event (American Psychiatric Association APA 2013). Most people experience at least one traumatic event in their lifetime, and approximately 8.3% of individuals who have been exposed to a traumatic event will develop PTSD (Kessler et al. 2017; Kilpatrick et al. 2013). PTSD is associated with functional impairment (Bovin et al. 2018; Jellestad et al. 2021), as well as increased risk for a variety of maladaptive health‐risk behaviors (e.g., substance use, eating pathology, nonsuicidal self‐injury; Contractor et al. 2019), including suicidal thoughts and behaviors (STB; Ford and Gómez 2015; Kobrinsky and Siedlecki 2024; Panagioti et al. 2012).

Additionally, even individuals who endorse some PTSD symptoms but do not meet full DSM‐5 diagnostic criteria for PTSD are at elevated risk for negative outcomes (Korte et al. 2016). Specifically, subthreshold PTSD is associated with distress, poorer interpersonal functioning, STB, and greater risk for the development of clinical‐level PTSD symptoms (Cukor et al. 2010; Marshall et al. 2001; Mylle and Maes 2004). In a nationally representative sample of adults with PTSD in the United States, the lifetime rates of suicidal ideation and suicide attempts were 31.9% and 13.6%, respectively (LeBouthillier et al. 2015)—higher than rates observed among individuals without PTSD (15.6% and 0.7% respectively; Strashny et al. 2023; Substance Abuse and Mental Health Services Administration SAMHSA 2022). Given the robust association between PTSD symptoms and STB, there is benefit in identifying malleable factors that may influence this association. Such research could inform the development of targeted STB interventions among individuals who experience PTSD symptoms.

Studies have consistently shown that individuals who have been exposed to a traumatic event and/or have PTSD symptoms exhibit heightened difficulties with emotion regulation (McLean and Foa 2017; Tull et al. 2020). These heightened difficulties with emotion regulation may also place individuals with PTSD symptoms at elevated risk for STB (Rogante et al. 2024; Turton et al. 2021). Studies have shown that difficulties in emotion regulation are associated with suicidal ideation in clinical samples (Law et al. 2015; Rogante et al. 2024; Turton et al. 2021), and there is some evidence (e.g., Denning et al. 2022; Kobrinsky and Siedlecki 2024) supporting a link between difficulties in emotion regulation and suicidal behaviors (e.g., suicide planning or attempts) as well (although other studies have failed to find a significant association; see Harris et al. 2018). According to Anestis et al. (2014), the connection between emotion regulation difficulties and suicidal behavior may be accounted for by the use of maladaptive, self‐destructive behaviors to regulate emotions that also inadvertently increase capability for suicide (e.g., self‐injury; Anestis et al. 2014; Hamza et al. 2012; Martin et al. 2017).

In support of the role of emotion regulation difficulties in STB among individuals with PTSD symptoms, Kobrinsky and Siedlecki (2024) found that difficulties in emotion regulation accounted for the association between PTSD symptom severity and suicidal behaviors. Moreover, Martin et al. (2017) provided evidence that PTSD symptom severity is associated with more severe suicidal ideation among psychiatric inpatients reporting greater difficulties in emotion regulation. However, one aspect of emotion regulation that has received less attention to date in the association between PTSD and STB is the *perceived* ability to regulate emotions (or emotion regulation self‐efficacy [ERSE]; Caprara and Gerbino 2001).

Self‐efficacy theory posits that an individual's motivation for engaging in a behavior depends on their belief that the desired result will be achieved (Bandura 1997; Poluektova et al. 2023). The intense and frequent negative emotions associated with PTSD symptoms may make emotions more difficult to modulate, decreasing ERSE for some individuals with these symptoms. For example, some individuals with PTSD symptoms may find that emotion regulation strategies that were once effective in response to emotional distress are no longer sufficient for modulating the myriad intense negative emotions that coincide with PTSD symptoms. In addition, the increased need to regulate emotions among individuals with PTSD symptoms could deplete capacity (e.g., emotional and cognitive) for regulation (Tull et al. 2020). Consequently, some individuals with PTSD symptoms may experience a reduction in their perceived ability to effectively modulate their emotions—a factor that could contribute to increased helplessness, hopelessness, and entrapment (risk factors for suicide; O'Connor and Kirtley 2018). Moreover, as emotions are increasingly perceived as out of control or inescapable, individuals may turn to self‐destructive, emotionally‐avoidant strategies to escape their emotions in the moment (e.g., self‐injury) that have been found to increase capability for suicide (Hamza et al. 2012).

Notably, however, despite growing research documenting a negative association between PTSD symptoms and ERSE (e.g., Doolan et al. 2017; Mahoney et al. 2021; Mette et al. 2025; Shirley et al. 2022; Tull et al. 2020), little research has examined the role of ERSE in STB and no studies have examined the moderating role of ERSE in the relation between PTSD symptoms and STB. Nonetheless, the limited research available provides preliminary support for the relevance of ERSE to both STB and the relation of PTSD symptoms to STB. For example, several studies provide support for a negative association between ERSE and STB, finding that lower ERSE is associated with both suicidal ideation and suicidal behaviors (Kobrinsky and Siedlecki 2024; Raudales et al. 2023; Rogante et al. 2024; Spitzen et al. 2020). Additionally, there is preliminary evidence that ERSE may account for the relation between PTSD symptoms and STB (Kobrinsky and Siedlecki 2024; Raudales et al. 2023; Zeng et al. 2018). Finally, although not specific to PTSD symptoms, research supports the moderating role of ERSE in the relation between other suicide‐related risk factors and STB, with Liu et al. (2020) finding that greater ERSE buffered the association between emotion regulation difficulties and both suicidal ideation and engagement in non‐suicidal self‐injury in a sample of adolescents. As noted previously, however, no studies to date have examined whether ERSE moderates the association between PTSD severity and STB.

Thus, the goal of the present study was to extend extant research on the interrelations of ERSE, PTSD, and STB by examining the moderating role of ERSE in the association between PTSD symptom severity and STB within a community sample of individuals exposed to a criterion A traumatic event. We hypothesized that the relation between PTSD symptom severity and STB would be stronger for individuals with low or moderate ERSE, relative to those with high ERSE.

## 1 Methods

### 1.1 Participants

All participants (*N* = 227) for the current study were recruited via Amazon's Mechanical Turk. MTurk is an online crowdsourcing platform where participants complete surveys for monetary compensation. Studies have shown that data derived from MTurk are as reliable as data collected using other procedures (Buhrmester et al. 2011). Studies also show that MTurk samples have the advantage of being more diverse than other internet‐recruited or college student samples (Buhrmester et al. 2011; Casler et al. 2013) and tend to report more clinical symptoms (e.g., depression and anxiety) than non‐clinical samples recruited through other means (Arditte et al. 2016). To be included in the present study, participants were required to be at least 18 years of age, be residents of the United States, speak English fluently, have 95% or higher MTurk approval ratings, and report at least one PTSD Criterion A traumatic event. Participants also had to pass all validity checks within the surveys, which included the accurate completion of attention checks and reasonable completion times.

Participants for the current study (65.2% female sex at birth) ranged in age from 22 to 72 years (*M* = 39.91, SD = 11.75). As for racial/ethnic background, 85.5% self‐identified as White, 8.4% as Native American, 4.8% as Asian/Asian American, 3.1% as Latinx, 2.2% as Black/African American, and 1.8% as Middle Eastern/North African. Most participants (81%) reported having some college education and an annual income of >$40,000/year (63.4%). Participants most frequently reported experiencing natural disasters (*n* = 101; 44.5%), transportation accidents (*n* = 100; 44.1%), physical assaults (*n* = 76; 33.5%), and unwanted sexual experiences (*n* = 71; 31.3%); see Table 1 for additional frequencies. Most participants (*n* = 171; 75.3%) reported PTSD symptom levels below the recommended PCL‐5 cutoff for a probable PTSD diagnosis (≥33). In addition, most participants (*n* = 190; 83.7%) indicated that they never had a suicide attempt in their lifetime, with only 4.4% (*n* = 10) reporting one lifetime suicide attempt and 6.2% (*n* = 14) reporting two lifetime suicide attempts.

**Table 1:** Frequencies of reported experienced potentially traumatic events on the extended life events checklist for DSM‐5 (*N* = 227).

Event	*n*	%
Natural disaster	101	44.5%
Transportation accident	100	44.1%
Physical assault	76	33.5%
Other unwanted or uncomfortable sexual experience	71	31.3%
Sexual assault	60	26.4%
Any other stressful event/experience	60	26.4%
Life‐threatening illness or injury	35	15.4%
Assault with a weapon	27	11.9%
Fire or explosion	26	11.5%
Serious accident	25	11.0%
Captivity	12	5.3%
Exposure to toxic substance	11	4.8%
Severe human suffering	11	4.8%
Sudden violent death	5	2.2%
Sudden accidental death	5	2.2%
Combat or exposure to war‐zone	4	1.8%
Serious injury, harm, or death caused to someone else	4	1.8%

### 1.2 Measures

#### 1.2.1 Demographics

Participants responded to demographic questions, which asked about sex assigned at birth, gender, age, race/ethnicity, relationship status, and annual income.

#### 1.2.2 Trauma Exposure and PTSD Symptoms

Criterion A traumatic event exposure was assessed using the Extended Self‐Report Life Events Checklist for DSM‐5 (LEC‐5; Weathers, Blake, et al. 2013). On the Extended Self‐Report LEC‐5, participants indicate whether they experienced 17 different potentially traumatic events in their lifetime. Participants respond to each event by indicating if the event: happened to them directly, was witnessed, was learned about, occurred as part of their job, or did not apply. If an event was experienced, participants then complete a series of follow‐up questions designed to identify the traumatic event perceived to be the worst by participants (“Briefly describe the worst event [e.g., what happened, who was involved, etc.]”) and determine whether the event meets criterion A for PTSD (direct or indirect exposure to actual or threatened death, serious injury, or sexual violence). These follow‐up questions were reviewed by investigators to ensure that all worst events identified by participants met criterion A for PTSD.

Afterwards, participants completed the PTSD Checklist for DSM‐5 (PCL‐5; Weathers, Litz, et al. 2013) while referencing the worst event identified on the Extended Self‐Report LEC‐5. PCL‐5 scores were calculated only for participants who reported a traumatic event that met criterion A on the Extended Self‐Report LEC‐5. The PCL‐5 is a 20‐item, self‐report measure that assesses the current (past 30 days) experience of 20 DSM‐5 symptoms of PTSD. Participants rate each item on a 5‐point Likert‐type scale ranging from 0 (*not at all*) to 4 (*extremely*) to indicate how much they were bothered by each symptom in the past month. Responses were summed to create an overall severity of current PTSD symptoms score (Cronbach's *α* = 0.96 in this sample).

#### 1.2.3 ERSE

The Regulatory Emotional Self‐Efficacy Scale (RESE; Caprara et al. 2008) is a 12‐item, self‐report scale that was used to measure participants' beliefs regarding their ability to regulate negative emotions (e.g., “how well can you get over irritation quickly for wrongs you have experienced?”) and express positive emotions (e.g., “how well can you express joy when good things happen to you?”). Participants answer each item using a scale from 1 (*not well at all*) to 5 (*very well*). Higher scores reflect greater perceived self‐efficacy for the expression of positive emotions and management of negative emotions. This study focused only on the perceived self‐efficacy for managing negative emotions subscale (Cronbach's *α* = 0.91 in this sample). The RESE has demonstrated convergent validity with other constructs that would be expected to be related to positive and negative emotion regulation, such as self‐esteem, positive affect, prosocial behavior, negative affect, and anxiety/depression (Caprara et al. 2008).

#### 1.2.4 STB

Frequency and intensity of suicidal thoughts, plans, and impulses in the past 2 weeks were measured using the 4‐item Depressive Symptom Index‐Suicidality Subscale (DSI‐SS; Metalsky and Joiner 1997). For each item, participants select one of four statements. For example, for the first item on the measure, answer options range from “I do not have thoughts of killing myself” to “I always have thoughts of killing myself.” Responses to each item are assigned a score of 0 to 3. Higher scores reflect greater suicidal thoughts, plans, or impulses in the past 2 weeks (Cronbach's *α* = 0.93 in this sample). Research has shown that higher DSI‐SS scores are associated with depression symptoms and suicide attempt histories, and the DSI‐SS is widely used in research and clinical settings as a reliable and valid measure of suicidality (Capron et al. 2012; Cukrowicz et al. 2011; Joiner et al. 2002; Stanley et al. 2021). A cut‐off score of ≥3 has been used to classify individuals as at high risk for suicide (Joiner et al. 2002).

#### 1.2.5 Depression

The 12‐item depressive symptoms subscale of the Mood and Anxiety Symptom Questionnaire Short Form (MASQ‐SF; Clark and Watson 1991; Watson et al. 1995) was used to measure past‐week depression symptoms. This variable was included as a covariate in the primary analysis. Participants rate how much they experienced symptoms of depression, such as “felt sad” and “felt worthless,” using a 5‐point Likert‐type scale ranging from 1 (*very slightly or not at all*) to 5 (*extremely*). Higher scores indicate more severe depressive symptoms (Cronbach's *α* = 0.95 in this sample). The MASQ‐SF has demonstrated reliability, as well as convergent and discriminant validity, in adult samples (Liu et al. 2015; Watson et al. 1995).

### 1.3 Procedure

All procedures were approved by the institution's Institutional Review Board. Participants first responded to a Completely Automatic Public Turing test to Tell Computers and Humans Apart (CAPTCHA) before providing informed consent. Participants were also informed on the consent form that the study included a number of safeguards to ensure that participants provided valid and accurate data, and that failure to pass these attention and validity checks would result in their responses not being approved or paid and their data being discarded. Data were collected in blocks of nine participants at a time and all data, including attention check items and geolocations (i.e., geographical coordinates used to identify participants outside of the US and/or in locations determined to be “bot farms” within the MTurk community; see Kennedy et al. 2020), were examined by researchers before compensation was provided. Participants were provided with a list of mental health and crisis resources after completing the surveys. Participants who failed one or more attention check items were removed from the study (*n* = 150 of 515 completers of the survey). Those who completed the survey and whose data were considered valid (based on attention check items and geolocation verification; *N* = 365) were compensated $3.00 for their participation. Of those 365 participants, 227 (the current sample) were determined to have experienced a criterion A traumatic event (62%, a rate consistent with that found in epidemiological studies; Mills et al. 2011).

### 1.4 Analysis Plan

Descriptive statistics for the primary variables of interest were computed, and skewness and kurtosis were evaluated (and corrected as needed). Pearson product‐moment correlations were conducted to evaluate zero‐order associations among variables. Next, a hierarchical linear regression analysis was conducted examining the main and interactive associations of PTSD symptom severity and perceived self‐efficacy for managing negative emotions with STB. Depressive symptom severity was included as a covariate in the first step of the model, given the high co‐occurrence of depression with both PTSD symptoms (Flory and Yehuda 2015; Nock et al. 2010) and STB (Klonsky et al. 2016). Indeed, depressive symptom severity was found to be significantly positively associated with all primary variables of interest (|*r* | s > 0.43, *p* < 0.001). Moreover, given evidence of sex and age differences in STB (Fox et al. 2018; Rossom et al. 2017), we also explored whether these demographic variables required inclusion in our model. Although sex assigned at birth was not significantly associated with STB (*r* = −0.02, *p* = 0.720), age was significantly negatively associated with STB (*r* = −0.18, *p* = 0.006). Thus, age was also included as a covariate in the first step of our model. PTSD symptom severity and perceived self‐efficacy for managing negative emotions (both mean centered) were entered in the second step of the model, followed by the product of these variables in the third step. The PROCESS macro version 4.2 for SPSS (Hayes 2012) was used to probe significant interactions by examining simple slopes representing the association between PTSD symptom severity and STB as a function of the perceived ability to manage negative emotions (plotted at standard values of −1 SD, mean, +1 SD).

## 2 Results

### 2.1 Preliminary Analyses

Table 2 presents descriptive data and correlations among variables. According to the standard cutoff on the DSI‐SS used to classify individuals as high‐risk for a future suicide attempt (i.e., scores ≥ 3; Joiner et al. 2002), 14.5% of the sample met or exceeded this cutoff. All variables fell within the acceptable range of normality (see Byrne 2010), with the exception of the DSI‐SS (skewness = 2.75, kurtosis = 7.77). Following log‐transformation, the DSI‐SS approximated a normal distribution (skewness = 2.24, kurtosis = 3.99). Subsequent analyses used the transformed DSI‐SS variable. PTSD symptom severity was significantly negatively associated with perceived self‐efficacy for managing negative emotions and significantly positively associated with STB. Perceived self‐efficacy for managing negative emotions was significantly negatively associated with STB.

**Table 2:** Means, standard deviations, and bivariate correlations between study variables.

Variable	1	2	3	4
1. Depressive symptom severity	—	0.590 (< 0.001)	0.440 (< 0.001)	0.532 (< 0.001)
2. PTSD symptom severity^a^		—	−0.178 (0.007)	0.431 (< 0.001)
3. ERSE‐negative			—	−0.228 (< 0.001)
4. STB				—
Mean	24.51	19.67	25.28	0.86
Median	20.00	13.00	25.00	0.00
Mode	12.00	0.00	23.00	0.00
Standard deviation	12.68	19.18	7.61	2.07
Range	12−60	0−80	12−60	0−12

*Note: p* value are presented in parentheses next to correlation statistic. Correlations for STB were conducted using transformed scores. Descriptive statistics for STB used non‐transformed data to aid in interpretability.

Abbreviations: ERSE‐Negative = perceived self‐efficacy for managing negative emotions, PTSD = post‐traumatic stress disorder, STB = Suicidal thoughts and behaviors.

^a^ Descriptive statistics for PTSD symptom clusters: (a) Intrusions: Mean = 4.85, SD = 5.27, Range = 0−20; (b) Avoidance: Mean = 2.68, SD = 2.53, Range = 0−8; (c) Cognition and Mood Alterations: Mean = 6.75, SD = 7.49, Range = 0−28; (d) Hyperarousal: Mean = 5.40, SD = 5.92, Range = 0−24.

### 2.2 Primary Analysis

The overall model was significant and accounted for 33% of the variance in STB (*F* [4, 222] = 21.69, *p* < 0.001; see Table 3). PTSD symptom severity and perceived self‐efficacy for managing negative emotions explained an additional 2% (*F* [2, 222] = 3.20, *p* = 0.042) of the variance in STB above and beyond depressive symptom severity. Whereas PTSD symptom severity was significantly uniquely associated with STB (*b* = 0.001, SE = 0.0004, *p* = 0.012), perceived self‐efficacy for managing negative emotions was not significantly associated with STB (*b* = ‐.0002, SE = 0.0009, *p* = 0.825).

**Table 3:** Main and interactive effects of PTSD symptom severity and perceived self‐efficacy for managing negative emotions on suicidal thoughts and behaviors.

	*R* ^2^	*ΔR* ^2^	*F*	*b*	SE	*p*
*Step 1*	0.28	0.29^a^	*F*(2, 224) = 45.10*			
Depression symptom severity				0.0046	0.0005	< 0.001
Age				−0.0006	0.0006	0.269
*Step 2*	0.30	0.02^b^	*F*(4, 222) = 24.59*			
Depression symptom severity				0.0037	0.0007	< 0.001
Age				−0.0005	0.0006	0.343
PTSD symptom severity				0.0010	0.0004	0.012
ERSE‐Negative				−0.0002	0.0009	0.825
*Step 3*	0.31	0.02^c^	*F*(5, 221) = 21.69*			
Depression symptom severity				0.0037	0.0007	<0.001
Age				−0.0005	0.0006	0.354
PTSD symptom severity				0.0010	0.0004	0.014
ERSE‐negative				−0.0005	0.0009	0.604
PTSD X ERSE				−0.0001	0.00004	0.007

Abbreviations: ERSE‐Negative = perceived self‐efficacy for managing negative emotions, PTSD = post‐traumatic stress disorder.

^a^ Δ*F* = *F* (2, 224) = 45.10, *p* < 0.001.

^b^ Δ*F* = *F* (2, 222) = 3.20, *p* = 0.042.

^c^ Δ*F* = *F* (1, 221) = 7.29, *p* = 0.007.

**Figure 1:**
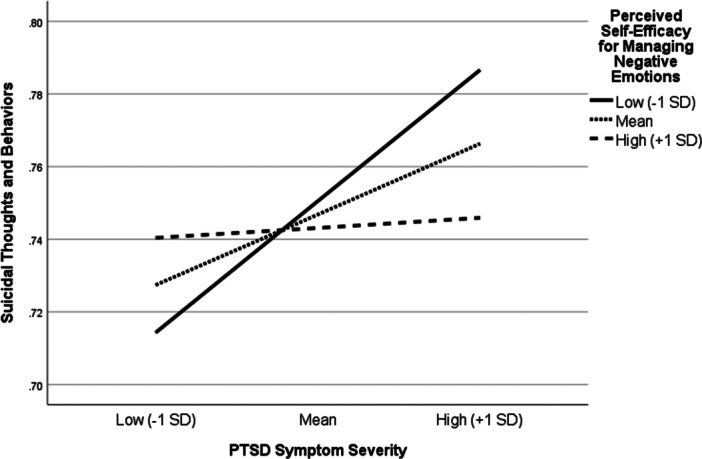
Perceived self‐efficacy for managing negative emotions moderates the association between PTSD symptom severity and suicidal thoughts and behaviors.

The addition of the interaction term significantly improved the model, explaining an additional 3.3% of the variance in STB (*F* [1, 222] = 7.29, *p* = 0.007). As expected, there was a significant interaction between PTSD symptom severity and perceived self‐efficacy for managing negative emotions (*b* = −0.0001, SE = 0.00004, *p* = 0.007). Evaluation of simple slopes indicated significant positive associations between PTSD symptom severity and STB at low (*b* = 0.0019, SE = 0.0005, *p* = 0.0003, 95% CI [0.0009, 0.0029]) and moderate (*b* = 0.0010, SE = 0.0004, *p* = 0.014, 95% CI [0.0002, 0.0018]), but not high (*b* = 0.0001, SE = 0.0005, *p* = 0.783, 95% CI [−0.0009, 0.0012], levels of perceived self‐efficacy for managing negative emotions (see Figure 1).

## 3 Discussion

This study aimed to explore whether perceived self‐efficacy for the regulation of negative emotions influenced the strength of the association between PTSD symptom severity and STB within a community sample of individuals exposed to a criterion A traumatic event. Results provided preliminary support for hypotheses, as we found a significant positive association between PTSD symptom severity and STB only among individuals who reported low or moderate levels of ERSE. Given that PTSD symptom severity was not significantly associated with STB among individuals with high ERSE, ERSE may operate as a protective factor for STB among individuals exposed to a traumatic event and experiencing PTSD symptoms.

These initial results can be interpreted through an avoidance and emotional mastery lens. The intense and frequent negative emotions (e.g., shame, guilt, fear, and anger), thoughts, and memories associated with PTSD may contribute to increased STB, which serve an experientially avoidant function by offering an escape from aversive experiences that are perceived as intolerable (Hayes et al. 1996). The desire to escape internal experiences may be particularly high among individuals who feel trapped by these experiences and doubt their ability to modulate their emotions. Alternatively, individuals who believe that they can effectively modulate their emotions may experience a greater sense of mastery over emotion regulation, increasing their ability to tolerate and modulate aversive internal states and thus decreasing contemplation of suicide as a way to escape these states (O'Connor and Kirtley 2018).

Although promising, findings are preliminary and must be interpreted in the context of study limitations. First, data were obtained through self‐report measures and were cross‐sectional in nature, introducing the potential for social desirability and recall biases and interfering with determining the precise direction of the examined relations. In addition, although data were examined to ensure that participants reported a valid criterion A event and participants were instructed to refer to that event when reporting the severity of their PTSD symptoms, it is possible that participants did not follow these instructions or were hesitant to report their most distressing event. Future studies would benefit from the use of diagnostic interviews to assess PTSD symptoms. Likewise, although the DSI‐SS has been found to be a reliable and valid assessment of STB (Batterham et al. 2015; Stanley et al. 2021), there is some evidence that clinical interviews may provide a more accurate assessment of STB (Lungu et al. 2019). That said, other studies have shown a high level of agreement between self‐report and clinical interview assessments of STB (Kaplan et al. 1994). Regardless, replication of the current findings using more rigorous and in‐depth assessments of the primary variables of interest is needed.

In addition, whereas the specific model tested here was theoretically‐derived, the precise nature and direction of the relations examined here may be different than proposed. For example, elevated STB combined with low ERSE may contribute to the worsening of some PTSD symptoms (e.g., helplessness, decreased positive affect, increased feelings of isolation, or detachment). Alternatively, the experience of severe PTSD symptoms combined with elevated STB may reduce confidence in one's ability to effectively regulate negative emotions. Future studies examining the interrelations of PTSD symptoms, ERSE, and STB over time (e.g., through ecological momentary assessment methods) are needed to clarify the precise interrelations and directions of the variables explored in this model.

Additionally, our sample introduces limitations related to the broader generalizability of our findings to the larger U.S. adult population and individuals with more severe PTSD symptoms (including clinical samples of patients with PTSD). Although previous research has shown that MTurk can aid in the recruitment of more diverse samples, this was not the case with our sample, which was primarily White and female (thereby limiting generalizability of our findings to the broader population). Future studies would benefit from targeting populations that are typically underrepresented in research and found to be at heightened risk for STB (e.g., racial/ethnic, gender, and sexual minoritized individuals). These sample limitations notwithstanding, it is notable that we were able to recruit a clinically relevant sample, with approximately 25% of participants meeting criteria for a probable diagnosis of PTSD and 15% reporting STB indicative of high risk for a future suicide attempt. Moreover, participants endorsed exposure to a variety of traumatic events (which aids in generalizing results to other trauma‐exposed populations), including events that have a high likelihood of contributing to PTSD symptoms (e.g., interpersonal violence; Lukaschek et al. 2013). Finally, although we were able to detect a significant moderation effect, the observed interaction accounted for only a modest amount of variance in STB. Replication of these findings in larger, more diverse, and clinical samples is needed. Future research would also benefit from considering the role of other variables that may interact with ERSE to predict STB.

Despite the aforementioned limitations, these preliminary results add to the growing body of literature on the interrelations of PTSD, ERSE, and STB (e.g., Gratz et al. 2020; Liu et al. 2020; Zeng et al. 2018) and provide preliminary support for the relevance of ERSE to the association between PTSD symptoms and STB. Indeed, despite growing research on ERSE, this novel facet of emotion regulation remains understudied, especially with regard to its associations with clinical phenomena. Although research has already begun to highlight the need for suicide interventions to target emotional self‐efficacy in the prevention of suicidal ideation (Liu et al. 2020), future studies with more rigorous designs (e.g., prospective studies that explore the unique role of ERSE in the development or maintenance of various forms of psychopathology and maladaptive behaviors) are needed. Such research may ultimately inform the development of novel clinical interventions for STB and other self‐destructive behaviors among high‐risk populations. Although some interventions currently include emotion regulation as a treatment target (e.g., Dialectical Behavioral Therapy [Linehan 1993], Acceptance‐based Emotion Regulation Therapy [Gratz and Tull 2025]), their effect on ERSE, PTSD symptoms, and STB specifically requires exploration.

## Ethics Statement

All procedures related to this manuscript were approved by the relevant Institutional Review Board.

## Consent

All participants accessed an electronic IRB‐approved letter of invitation and provided consent before continuing to study items. Anonymity of participant identity was assured.

## Conflicts of Interest

The authors declare no conflicts of interest.

## Data Availability

The data that support the findings of this study are available from the corresponding author upon reasonable request.
